# A crystal-processing machine using a deep-ultraviolet laser: application to long-wavelength native SAD experiments

**DOI:** 10.1107/S2053230X2101339X

**Published:** 2022-01-27

**Authors:** Yoshiaki Kawano, Masahide Hikita, Naohiro Matsugaki, Masaki Yamamoto, Toshiya Senda

**Affiliations:** a Advanced Photon Technology Division, RIKEN SPring-8 Center, 1-1-1 Kouto, Sayo-cho, Sayo-gun, Hyogo 679-5198, Japan; b Structural Biology Research Center, Institute of Materials Structure Science, High Energy Accelerator Research Organization (KEK), 1-1 Oho, Tsukuba, Ibaraki 305-0801, Japan

**Keywords:** X-ray crystallography, deep-UV laser, native SAD phasing, crystal processing

## Abstract

While native SAD phasing is a promising method for next-generation macromolecular crystallography, it requires the collection of high-quality diffraction data using long-wavelength X-rays. The crystal itself and the noncrystalline medium around the crystal can cause background noise during long-wavelength X-ray data collection, hampering native SAD phasing. Optimizing the crystal size and shape or removing noncrystalline sample portions have thus been considered to be effective means of improving the data quality. A crystal-processing machine that uses a deep-UV laser has been developed. The machine utilizes the pulsed UV laser soft ablation (PULSA) technique, which generates less heat than methods using infrared or visible lasers. Since protein crystals are sensitive to heat damage, PULSA is an appropriate method to process them. Integration of a high-speed Galvano scanner and a high-precision goniometer enables protein crystals to be shaped precisely and efficiently. Application of this crystal-processing machine to a long-wavelength X-ray diffraction experiment significantly improved the diffraction data quality and thereby increased the success rate in experimental phasing using anomalous diffraction from atoms.

## Abstract 2

A crystal-processing machine that uses a deep-ultraviolet laser has been developed. The machine can improve diffraction data by optimizing the crystal size and shape or by removing unnecessary portions of the crystal.

## 1. Introduction

X-ray crystallography is one of the primary methods used for the three-dimensional structure determination of proteins at atomic resolution. The protein crystals to be analyzed are usually mounted on a cryoloop with crystallization solution and then cooled, typically with liquid nitrogen. The cooled crystals are then fixed on a goniometer and used for X-ray diffraction data collection. However, scattering from the cryoloop and from the crystallization solution around the crystal causes background noise, which degrades the data quality. In recent years, it has widely become possible to determine phases using the native single anomalous diffraction (SAD) method, which utilizes anomalous diffraction from S atoms in the protein. Since the method does not require the introduction of heavy atoms into the protein crystal for phasing, it could become a next-generation phasing method in protein crystallography (Liu *et al.*, 2012 ▸; Weinert *et al.*, 2015 ▸; Guo *et al.*, 2019 ▸). However, a specific strategy is needed to obtain high-quality diffraction data because the long-wavelength X-rays used in the native SAD method are easily affected by the solvent, the cryoloop and the size and shape of the crystal (Liebschner *et al.*, 2016 ▸; Basu *et al.*, 2019 ▸). Several methods have been developed to reduce background noise: mounting crystals using adhesive materials (Kitatani *et al.*, 2009 ▸), a graphene membrane (Wierman *et al.*, 2013 ▸) or a mesh loop (Pellegrini *et al.*, 2011 ▸). Notably, the capillary-top mount (also called the loopless mount) method (Kitago *et al.*, 2005 ▸) has been improved and successfully applied to native SAD phasing with long-wavelength X-rays (Watanabe, 2006 ▸; Kitago *et al.*, 2010 ▸; Yu *et al.*, 2020 ▸). However, all of the above methods require elaborate sample manipulation and cannot change the size or shape of the crystal, which has been considered to be another source of experimental error.

To reduce the errors originating from a mounted crystal, it is necessary to remove the unnecessary portions of the crystal and process the remainder into the desired shape. For these purposes, a laser-processing technique would be ideal. Laser processing has mainly been utilized for fabricating metals and plastics and is based on thermal processes using infrared (or visible) light sources, such as a CO_2_ laser (wavelength 10.6 µm) or an Nd:YAG laser (wavelength 1060 nm). However, since protein crystals are hypersensitive to changes in temperature and humidity (McPherson, 1999 ▸; Tachibana *et al.*, 1999 ▸), it is difficult to apply these lasers to protein crystals. Thus, we considered processing based on deep-UV laser ablation. In general, the ablation threshold (the minimum laser fluence required for processing) depends on the absorbance of the target. Proteins have a broad absorption spectrum below a wavelength of 300 nm. It is known that the absorption coefficient below 200 nm is more than 20 times larger than that at 280 nm (Goldfarb *et al.*, 1951 ▸; Mayer & Miller, 1970 ▸). UV pulses with high absorbance at the crystal surface enable photoablation with low power consumption, which is desirable to avoid unnecessary heating. Moreover, since photoablation is limited to the irradiated crystal surface, precise control of the laser-irradiation area is possible. Therefore, a 193 nm pulsed deep-UV laser is employed as the light source for crystal processing. In a technique called pulsed UV laser soft ablation (PULSA), crystals can be processed gently by repeated irradiation with deep-UV light pulses (Kitano *et al.*, 2004*a* ▸,*b* ▸; Murakami *et al.*, 2004 ▸; Kitano, Matsumura *et al.*, 2005 ▸; Kitano, Murakami *et al.*, 2005 ▸).

Although crystal processing using PULSA was originally developed to extract a good portion of a sample crystal, it is also quite useful for removing unnecessary parts of the crystal for long-wavelength X-ray experiments. Here, we report a crystal-processing machine using a deep-UV laser for efficient sample fabrication and demonstrate native SAD experiments as a successful application.

## 2. Device and usage

**Figure 1:**
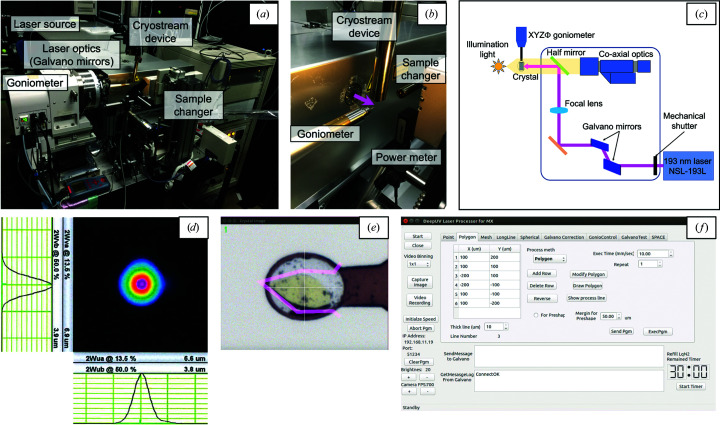
(*a*) Overall and (*b*) close-up views of the crystal-processing machine. The laser beam path is shown in pink. (*c*) A schematic representation of the device configuration and the laser optics. (*d*) The 2D Gaussian beam profile for the deep-UV laser. (*e*) A sample image from the co-axial viewing system. (*f*) GUI of the control software for laser irradiation.

The crystal-processing machine integrates a laser source, focusing optics with a Galvano scanner, a goniometer with a co-axial viewing camera to align the sample, a cryostream device and a sample-exchange robot (Fig. 1 ▸ *a*).

### 2.1. Laser source

The laser oscillator used for the crystal-processing machine is an NSL-193L (Nikon Co). This device emits a laser beam of 193 nm wavelength with a pulse width of less than 1.3 ns and a frequency of 1–400 kHz. The maximum pulse intensity is 1.0 µJ per pulse, which was upgraded from the original 0.25 µJ per pulse. As the NSL-193L is a class IV laser, it is placed in a booth with an interlock system. This device is equipped with a wavelength-conversion crystal to generate a laser beam of 193 nm wavelength. Since the conversion crystal is highly sensitive to humidity, continuous temperature control and a N_2_ gas purge are needed. However, constant deterioration of the wavelength-conversion crystal is inevitable. Therefore, the laser power downstream of the sample point is regularly monitored using a power meter (PD300-UV-193; Ophir Optronics Solutions) to check the deterioration (Fig. 1 ▸ *b*).

### 2.2. Laser optics

The laser optics of the crystal-processing machine are designed to allow high-speed movement of the laser-irradiation position while keeping the laser focal size unchanged in the scanning plane. Two Galvano mirrors control the beam direction of the deep-UV laser, which is changed by 90° at a mirror placed just upstream of the sample position (Fig. 1 ▸ *c*). A focusing lens focuses the laser beam to 6.6 × 6.9 µm (horizontal × vertical, FWHM) on the sample position (Fig. 1 ▸ *d*). While a previous system had a spot diameter of 15 µm (Kitano, Matsumura *et al.*, 2005 ▸), the laser focal size has been reduced to less than half that in the previous system by the improvement in laser optics. In addition, the laser fluence has been increased from 0.1 to 8.0 J cm^−2^ compared with the previous system (Kitano, Matsumura *et al.*, 2005 ▸). The half mirror only reflects light of wavelength around 193 nm (Fig. 1 ▸ *c*). Since the mirror transmits light of other wavelengths, the sample can be observed from the same direction as the laser using a co-axial viewing camera (Fig. 1 ▸ *e*).

### 2.3. Sample holding and manipulation

A goniometer has four axes (*X*, *Y*, *Z* and φ, the rotation axis) to adjust the sample position and orientation during processing. An air-bearing goniometer (AB-150R; Canon) was adopted for the rotation axis to keep the eccentricity accuracy to within 1 µm. A cryostream device is used to keep the sample cooled during crystal processing. The SPACE robot (Murakami *et al.*, 2012 ▸) was introduced for efficient and reliable sample exchange with a storage Dewar into which up to two UniPucks can be loaded.

### 2.4. Control software

The control software (Fig. 1 ▸ *f*), written in Qt4 and GCC, has a registration panel for the vertex positions of the polygon on the scanning plane. To process the sample, the laser beam is moved along the polygon lines at a specific speed (typically 10 mm s^−1^). The polygon shape can be changed directly on the control monitor by using a mouse to drag the vertices on the mounted crystal image (Fig. 1 ▸ *e*). The width of the polygon lines and the number of laser-irradiation repetitions can be specified, and can be increased for samples that are difficult to process. It is possible to change the shape to an φ-axis-symmetric rotational body such as a sphere or cylinder by creating a polygon in the scanning plane and then rotating the sample in a stepwise manner around the φ axis. The step angle and the total rotation angle for the shaping can be specified through the control software. In addition, the control software includes sample-alignment and sample-exchange functions. A sample can be processed remotely through the *NoMachine* software (NoMachine Co.).

### 2.5. Applications in protein crystals

**Figure 2:**
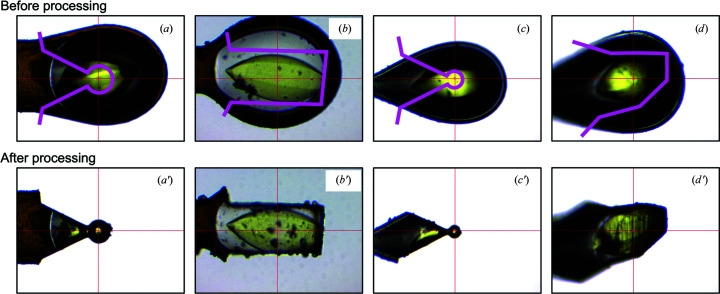
Examples of crystal processing. Crystals are shown before (*a*)–(*d*) and after (*a*′)–(*d*′) crystal processing. Crystals are on either a litholoop (*a*, *b*) or a nylon loop (*c*, *d*). Crystals were spherically processed in (*a*′) and (*c*′) and the solvent was removed in (*b*′) and (*d*′). The laser scanning paths are shown by pink lines.

Since a deep-UV laser can process protein crystals, the surrounding solvent and cryoloops that are made of polymers such as nylon, users can freely specify the processing shape, regardless of the constitution of the sample. Note that the damage to protein crystals caused by deep-UV laser irradiation is restricted to around a few micrometres from the footprint of the laser beam, as previously reported (Basu *et al.*, 2019 ▸). According to the purpose of the crystal processing, users can choose an appropriate processing mode. There are two typical processing modes: solvent-removal processing and spherical processing (Fig. 2 ▸). In solvent-removal processing, users can selectively remove the solvent part, cryoloops and undesirable portions of multicrystals. In spherical processing, the sample is repeatedly irradiated by the deep-UV laser and rotated by the goniometer to form a sphere or cylinder. A movie showing an example of crystal processing is available in the supporting information.

A typical workflow for the crystal-processing experiments is as follows. Firstly, the crystal quality and the position in the cryoloop are evaluated by X-ray raster scanning using a routine function of the *PReMo* (*Photon Factory Remote Monitoring System*) data-collection software (Yamada *et al.*, 2013 ▸). This step is required because crystals with thin plate- or needle-like shapes or those covered with frost are difficult to recognize visually on the control monitor, and thus the quality of the crystal can only be evaluated from diffraction. When the crystal quality differs among regions of the crystal, the high-quality region of the crystal should be reserved for raster scanning-based processing. After checking the position and quality of the crystal, the crystal is mounted on the processing machine and processed by referring to the results of X-ray raster scanning. Finally, the processed crystal is mounted on the diffractometer of a beamline for diffraction data collection. The crystal-processing machine can store two UniPucks and can process up to 32 crystals at one time.

## 3. Native SAD experiments

As an example of a crystal-processing application, long-wavelength native SAD experiments were carried out. The data statistics and the success rates of phasing trials with *SHELXD* were examined using data before and after crystal processing. An NADH-dependent ferredoxin reductase, BphA4, from *Acidovorax* sp. strain KKS102 was used as a reference crystal (Senda *et al.*, 2000 ▸). In addition, crystals of carbonic anhydrase from *Anabaena* sp. PCC7120 (All2909; Hirakawa *et al.*, 2021 ▸) were used as a challenging target for native SAD phasing. All previous native SAD trials with unprocessed carbonic anhydrase crystals had failed, probably due to the low sulfur content.

### 3.1. Methods

BphA4 is a protein with 408 amino-acid residues containing nine S atoms, from five methionine and four cysteine residues, and two P atoms in flavin adenine dinucleotide (FAD). Crystals were prepared as described previously (Senda *et al.*, 2000 ▸). Native SAD data were collected using an X-ray wavelength of 2.7 Å on beamline BL-1A of the Photon Factory, KEK, Tsukuba, Japan (KEK-PF). BL-1A is specifically designed for long-wavelength X-ray diffraction experiments using protein crystals. The experimental conditions with low dose (approximately 1 MGy per data set) are shown in Table 1 ▸. To confirm that the radiation damage was negligible, we collected diffraction data sets three times from each crystal before and after laser processing.

**Figure 3:**
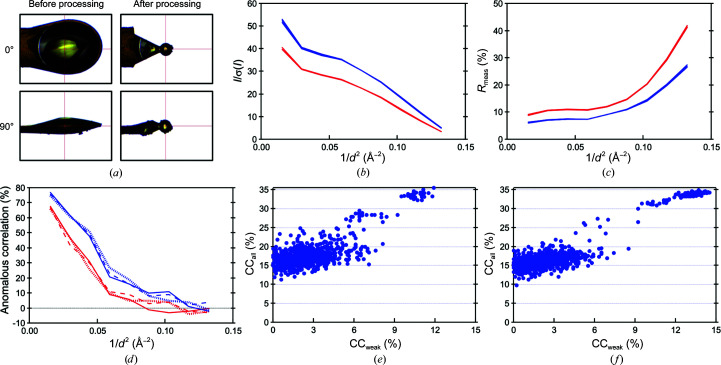
Comparison of the diffraction data quality of a BphA4 crystal before and after spherical processing. (*a*) A crystal before and after spherical processing. (*b*)–(*d*) *I*/σ(*I*), *R*_meas_ and anomalous correlation values are plotted against the diffraction resolution. Data sets 1, 2 and 3 were collected before processing (in red) and data sets 4, 5 and 6 were collected after processing (in blue) (see Table 2 ▸). In (*b*) and (*c*), the lines for data sets 1–3 and those for data sets 4–6 are nearly entirely overlaid. In (*d*), data sets 1 and 4, data sets 2 and 5, and data sets 3 and 6 are plotted with solid, dotted and dashed lines, respectively. (*e*, *f*) The CC_all_–CC_weak_ scatter plot from the *SHELXD* calculations for a crystal before (*e*) and after (*f*) spherical processing.

**Figure 4:**
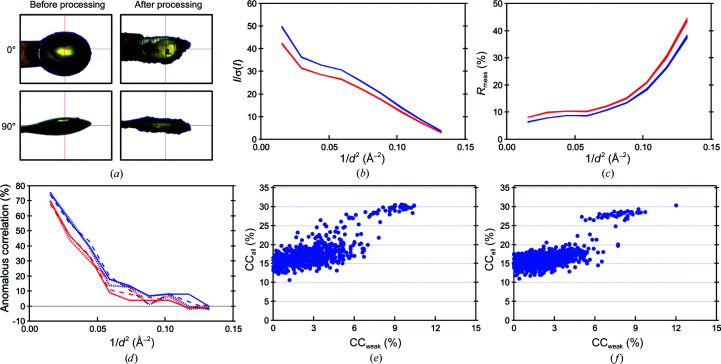
Comparison of the diffraction data quality of a BphA4 crystal before and after solvent-removal processing. (*a*) A crystal before and after solvent removal. (*b*)–(*d*) *I*/σ(*I*), *R*_meas_ and anomalous correlation values plotted against the diffraction resolution. Data sets 1, 2 and 3 were collected before processing (in red) and data sets 4, 5 and 6 were collected after processing (in blue) (see Table 3 ▸). In (*d*), data sets 1 and 4, data sets 2 and 5, and data sets 3 and 6 are plotted with solid, dotted and dashed lines, respectively. (*e*, *f*) The CC_all_–CC_weak_ scatter plot from the *SHELXD* calculations for a crystal before (*e*) and after (*f*) solvent-removal processing.

Spherical and solvent-removed BphA4 crystals were prepared to evaluate the effectiveness of crystal processing. The laser-irradiation conditions were 1.0 µJ per pulse at 5.0 kHz, with an average laser power of about 3.3 mW at the sample position. Shaping of a 500 × 300 × 150 µm crystal into a sphere with a diameter of 120 µm took about 20 min (Fig. 3 ▸ *a*), and solvent removal from a BphA4 crystal took about 10 min under the same laser-irradiation conditions (Fig. 4 ▸ *a*). All diffraction data were processed by *XDS* (Kabsch, 2010*a* ▸,*b* ▸), and the substructure was determined by *SHELXC*/*D* (Sheldrick, 2010 ▸) with the *HKL*2*MAP* interface (Pape & Schneider, 2004 ▸). *SHEXLD* calculations were performed using 1000 trials. We define a successful trial in the *SHELXD* calculation using two criteria. Firstly, the CC_all_–CC_weak_ scatter plot from the 1000-trial *SHELXD* calculation needs to be clearly split into two distinct distribution groups: high and low CC_all_/CC_weak_ groups. Secondly, the CC_all_ value needs to be greater than 30% in the high CC_all_/CC_weak_ group.

Carbonic anhydrase from *Anabaena* sp. PCC7120 (hereafter referred to as carbonic anhydrase All2909) is composed of 178 amino-acid residues and contains only one methionine S atom. Since carbonic anhydrase All2090 does not contain metal ions (Hirakawa *et al.*, 2021 ▸), there are no significant anomalous scatterers apart from the S atom. Crystals were prepared as described previously (Hirakawa *et al.*, 2021 ▸). We processed crystals into spherical shapes with diameters of 80–100 µm using the same processing conditions as for BphA4. The data-collection conditions are shown in Table 1 ▸. We collected four data sets from one crystal with different orientations using a mini-kappa goniometer. The data for the native SAD phasing were prepared by merging data sets using *XSCALE* (Kabsch, 2010*a* ▸,*b* ▸) to enhance the weak anomalous signals by highly redundant data.

### 3.2. Results

Comparison of the data statistics for the BphA4 crystals between before and after spherical processing revealed that the overall *I*/σ(*I*) and *R*_meas_ were improved from 22.6% and 13.7% to 31.8% and 9.7%, respectively. The anomalous correlation was increased from 7.2% to 19.0% (Fig. 3 ▸ and Table 2 ▸). A significant effect of the solvent removal was also observed; *I*/σ(*I*), *R*_meas_ and anomalous correlation were improved from 22.8%, 13.6% and 8.1% to 26.8%, 11.7% and 19.0%, respectively (Fig. 4 ▸ and Table 3 ▸).

The success rate of substructure determination by *SHELXD* increased after both spherical and solvent-removal processing (Figs. 3 ▸ *e*, 3 ▸ *f*, 4 ▸ *e* and 4 ▸ *f*). While some trials showed a CC_all_ of greater than 30% before solvent removal, no evident split was observed in the CC_all_–CC_weak_ scatter plot. After solvent removal, we found one successful trial among the 1000 trials (Fig. 4 ▸ *f*). The data from a spherically processed crystal showed a more apparent improvement (Fig. 3 ▸), resulting in an increase in the success rate from 33/1000 to 114/1000. The remarkable improvement after spherical processing was probably caused by the minimized error from the X-ray absorption by the crystal itself; the diffracted X-ray path length inside the spherical crystal becomes uniform in the case of the spherical crystals.

**Figure 5:**
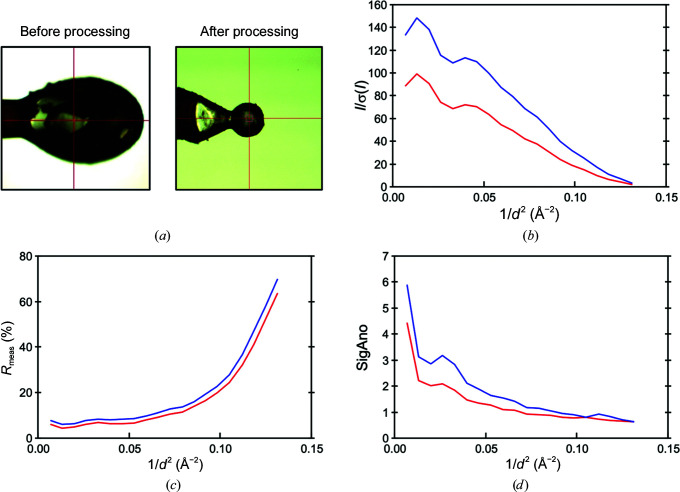
Diffraction data quality of spherically processed crystals of carbonic anhydrase All2909. (*a*) A crystal before and after spherical processing. (*b*)–(*d*) *I*/σ(*I*), *R*_meas_ and SigAno values plotted against the diffraction resolution. The graphs for data sets prepared by merging eight and 28 data sets are shown in red and blue, respectively.

Next, we examined the effect of the spherical processing using a challenging example: carbonic anhydrase All2909. This protein contains only one methionine residue, and the Bijvoet ratio was calculated as 0.86%. The data statistics of the merged data sets from spherically processed carbonic anhydrase All2909 crystals are shown in Fig. 5 ▸ and Table 4 ▸. Initially, native SAD phasing was performed using a data set with substantially high redundancy as a reference. This data set was prepared by merging 28 data sets from seven crystals. Since this data set was of high quality due to the high redundancy and low systematic errors of absorbance, native SAD phasing was possible. Next, the number of merged data was reduced to assess the effect of spherical shaping. We prepared another data set by merging eight data sets from only two crystals. Surprisingly, *autoSHARP* (Vonrhein *et al.*, 2007 ▸), which utilizes *SHELXD* for substructure determination, successfully solved the crystal structure by the native SAD method; 322 of the 356 residues of the dimeric molecule in the asymmetric unit were automatically built and sequenced, despite the very weak anomalous signal. This result demonstrates that dedicated data-collection environments with long-wavelength X-rays and the proper sample processing enable native SAD phasing even for challenging samples.

## 4. Conclusions and future prospects

We have developed a crystal-processing machine using a deep-UV laser to improve diffraction data quality with long-wavelength X-rays. The machine is quite helpful for native SAD data collection. We observed a significant improvement in diffraction data quality, probably due to a substantial reduction of background noise and errors from noncrystalline materials and anisotropic X-ray absorption by the crystal. Since practical details such as the optimization of crystal-processing conditions in native SAD experiments, including the effects of wavelength (a comparison of 2.7 and 3.3 Å) and sample size, have been reported previously (Basu *et al.*, 2019 ▸), we describe the configuration of the processing machine and control software in this report. In addition, systematic comparisons before and after crystal processing were performed using two proteins to clarify the effects of crystal processing.

In 2020, 11 167 structures were deposited in the Protein Data Bank (PDB), 530 of which were determined by SAD phasing. Most of the SAD phasing experiments used selenomethionine-substituted proteins. However, since the Bijvoet ratio (for native proteins) of almost all of the 530 proteins at 2.7 Å wavelength was higher than that of carbonic anhydrase All2909 (0.86%), our results suggest that these SAD structures in the PDB could have been solved by native SAD phasing, provided that the data collections were performed at a dedicated long-wavelength beamline with the proper treatment of samples to reduce errors of X-ray absorption.

Importantly, crystal processing using our machine is much easier than other techniques for reducing background noise and errors in long-wavelength X-ray data collection, because it does not require elaborate sample manipulation. Indeed, the crystal-processing machine can be operated by easy-to-use software with a graphical user interface (GUI), which allows users to operate the machine remotely. Furthermore, since the crystal-processing machine requires only a space of 2000 × 1200 mm for the surface plate, it can be integrated into most existing beamlines. Integration of the crystal-processing machine with the macromolecular crystallography beamline would be a critical step in the development of a macromolecular structure-solution pipeline using the native SAD method.

The crystal-processing machine can also be utilized for the microspectroscopy of protein crystals. In many cases, microspectroscopy is used as a complement to X-ray diffraction experiments using the same crystals. However, it is crucial to consider the thickness of a crystal when it is used in microspectroscopic experiments. It should be noted that the absorption spectrum of a thick crystal was difficult to measure due to significant light absorption by the crystal. Since the crystal size used for X-ray diffraction experiments is usually not suitable for microspectroscopic experiments, selecting a crystal with an appropriate size is necessary for microspectroscopy. In addition, the solvent around the crystal causes considerable background noise, resulting in a poor signal-to-noise ratio for the microspectroscopic data. Therefore, the solvent around the crystal should be removed, and the crystal thickness must be optimized to obtain a high-quality absorption spectrum. This device can solve both problems.

Notably, deep-UV laser processing can be applied not only to protein crystals but also to organic molecular crystals. The crystal-processing machine is also being used for time-resolved X-ray crystallography of small-molecule crystals that undergo structural changes triggered by laser irradiation. However, it is difficult to perform laser-irradiation experiments for the excitation of small-molecule crystals under the same conditions because of the different shapes and sizes of the crystals. Uniform laser irradiation is important in experimental systems that capture photoexcited structures. The crystal-processing machine enables us to process each crystal to nearly the same size and thus to conduct experiments using the same laser-irradiation conditions.

Upgrades to the control software are ongoing to integrate the crystal-processing software with *PReMo*, the data-management system for users of the macromolecular crystallography beamline at the Photon Factory (Yamada *et al.*, 2013 ▸). Users will be able to control the processing machine and beamline in an integrated environment and to monitor processing experiments from anywhere and access the experimental information at any later time through a web browser. In addition, users can perform processing experiments based on the results of the X-ray raster scanning previously recorded in the *PReMo* database. Integration of the databases for raster scanning and laser crystal processing will be helpful in cases with difficult crystal processing, such as the processing of small and frost-covered crystals. Furthermore, this integration will lead to automated crystal processing, which should be a part of the automated macromolecular crystallography pipeline.

## Supplementary Material

Captions to Supplementary Movies S1-S3. DOI: 10.1107/S2053230X2101339X/ow5030sup1.pdf

Supplementary Movie S1. DOI: 10.1107/S2053230X2101339X/ow5030sup2.mp4

Supplementary Movie S2. DOI: 10.1107/S2053230X2101339X/ow5030sup3.mp4

Supplementary Movie S3. DOI: 10.1107/S2053230X2101339X/ow5030sup4.mp4

## Figures and Tables

**Table 1:** Measurement conditions for long-wavelength X-ray diffraction experiments

Beamline	BL-1A, KEK-PF
Detector	EIGER X 4M
Wavelength (Å)	2.7000
Rotation range per image (°)	0.1
Total rotation range (°)	360
Exposure time per image (s)	0.01
Crystal-to-detector distance (mm)	60
Beam size (µm)	13 (horizontal) × 13 (vertical)
Beam shape	Gaussian

**Table:** Before crystal processing.

	Data set 1	Data set 2	Data set 3
Resolution range (Å)	49.19–2.76 (2.91–2.76)	49.19–2.76 (2.91–2.76)	49.20–2.76 (2.91–2.76)
No. of reflections	436467 (21685)	436351 (21614)	436471 (21509)
No. of unique reflections	13343 (1822)	13348 (1824)	13355 (1821)
Completeness (%)	99.6 (97.5)	99.6 (97.4)	99.6 (97.3)
*R*_merge_	0.137 (0.429)	0.138 (0.435)	0.138 (0.440)
〈*I*/σ(*I*)〉	22.6 (5.2)	22.5 (5.1)	22.5 (5.1)
CC_ano_	0.072 (−0.012)	0.067 (−0.007)	0.069 (−0.005)
Space group	*P*6_1_22	*P*6_1_22	*P*6_1_22
*a*, *b*, *c* (Å)	98.38, 98.38, 173.09	98.38, 98.38, 173.09	98.38, 98.38, 173.09

**Table:** After crystal processing.

	Data set 4	Data set 5	Data set 6
Resolution range (Å)	49.16–2.75 (2.90–2.75)	49.19–2.75 (2.90–2.75)	49.17–2.75 (2.90–2.75)
No. of reflections	434945 (20780)	435027 (20715)	434797 (20775)
No. of unique reflections	13371 (1805)	13376 (1803)	13380 (1807)
Completeness (%)	99.3 (95.2)	99.2 (94.9)	99.3 (95.1)
*R*_merge_	0.097 (0.279)	0.098 (0.279)	0.097 (0.284)
〈*I*/σ(*I*)〉	32.0 (7.2)	31.8 (7.0)	31.8 (6.9)
CC_ano_	0.190 (−0.044)	0.173 (−0.063)	0.199 (0.032)
Space group	*P*6_1_22	*P*6_1_22	*P*6_1_22
*a*, *b*, *c* (Å)	98.38, 98.38, 173.09	98.38, 98.38, 173.09	98.38, 98.38, 173.09

**Table:** Before crystal processing.

	Data set 1	Data set 2	Data set 3
Resolution range (Å)	49.25–2.76 (2.91–2.76)	49.25–2.76 (2.91–2.76)	49.26–2.75 (2.90–2.75)
No. of reflections	436686 (22297)	436738 (22208)	437143 (20757)
No. of unique reflections	13239 (1750)	13244 (1749)	13276 (1708)
Completeness (%)	99.0 (93.9)	99.0 (93.8)	98.6 (91.0)
*R*_merge_	0.136 (0.467)	0.136 (0.471)	0.137 (0.472)
〈*I*/σ(*I*)〉	22.8 (4.7)	22.7 (4.7)	22.5 (4.5)
CC_ano_	0.081 (−0.004)	0.078 (−0.016)	0.118 (0.014)
Space group	*P*6_1_22	*P*6_1_22	*P*6_1_22
*a*, *b*, *c* (Å)	98.50, 98.50, 172.54	98.50, 98.50, 172.58	98.50, 98.51, 172.62

**Table:** After crystal processing.

	Data set 4	Data set 5	Data set 6
Resolution range (Å)	49.25–2.76 (2.91–2.76)	49.26–2.76 (2.91–2.76)	49.26–2.76 (2.91–2.76)
No. of reflections	438213 (22037)	438363 (22047)	438178 (22126)
No. of unique reflections	13246 (1739)	13255 (1742)	13251 (1741)
Completeness (%)	99.0 (93.5)	99.0 (93.3)	98.9 (93.3)
*R*_merge_	0.116 (0.391)	0.117 (0.399)	0.117 (0.407)
〈*I*/σ(*I*)〉	26.8 (5.5)	26.7 (5.4)	26.6 (5.3)
CC_ano_	0.190 (0.029)	0.169 (−0.052)	0.166 (0.014)
Space group	*P*6_1_22	*P*6_1_22	*P*6_1_22
*a*, *b*, *c* (Å)	98.50, 98.50, 172.68	98.51, 98.51, 172.72	98.52, 98.52, 172.77

**Table 4:** Data-processing statistics for carbonic anhydrase All2909 crystals after spherical processing Values in parentheses are for the outer shell.

	Eight merged data sets from two crystals	28 merged data sets from seven crystals
Resolution range (Å)	49.25–2.76 (2.83–2.76)	49.25–2.76 (2.83–2.76)
No. of reflections	915930 (9469)	3192485 (34806)
No. of unique reflections	19331 (1204)	19501 (1354)
Completeness (%)	98.8 (83.8)	99.4 (92.4)
*R*_merge_	0.084 (0.639)	0.097 (0.700)
〈*I*/σ(*I*)〉	35.09 (1.40)	57.5 (3.20)
SigAno	1.055 (0.649)	1.358 (0.638)
Space group	*P*2_1_2_1_2_1_	*P*2_1_2_1_2_1_
*a*, *b*, *c* (Å)	53.191, 83.406, 88.501	53.191, 83.406, 88.501
