# Supplementary material for: A crystal-processing machine using a deep-ultraviolet laser: application to long-wavelength native SAD experiments
Source: Acta Crystallogr F Struct Biol Commun. 2022 Jan 27;78(Pt 2):88–95. doi: 10.1107/S2053230X2101339X (PMC8805218; doi:10.1107/S2053230X2101339X)
Supplement: Supplementary file 1 [file f-78-00088-sup1.pdf]

STRUCTURAL BIOLOGY COMMUNICATIONS

Volume 78 (2022)

Supporting information for article:

A crystal-processing machine using a deep-ultraviolet laser: application to long-wavelength native SAD experiments

Yoshiaki Kawano, Masahide Hikita, Naohiro Matsugaki, Masaki Yamamoto and Toshiya Senda

Movie S1 Demonstration of the processing of a BphA4 crystal into a sphere using a crystal processing machine. The crystal size was 400 × 250 × 100 μm^3 and the crystal was shaped into a sphere with a diameter of 90 μm. The deep-UV laser irradiation conditions were 1.0 μJ/pls at 5.0 kHz, with an average laser power at the sample position of about 3.3 mW.

Movie S2 Demonstration of the solvent removal processing of a BphA4 crystal using a crystal processing machine. The crystal size was 350 × 200 × 100 μm^3. The deep-UV laser irradiation conditions were 1.0 μJ/pls at 5.0 kHz, with an average laser power at the sample position of about 3.3 mW.

Movie S3 Demonstration of the processing of a BphA4 crystal into a cylinder using a crystal processing machine. The crystal size was 300 × 150 × 80 μm^3 and the crystal was shaped into a cylinder with a diameter of 70 μm. The deep-UV laser irradiation conditions were 1.0 μJ/pls at 5.0 kHz, with an average laser power at the sample position of about 3.3 mW.
